# Contact patterns and costs of multiple sclerosis in the Swedish healthcare system—A population‐based quantitative study

**DOI:** 10.1002/brb3.2582

**Published:** 2022-05-05

**Authors:** Jonas Lind, Sofia Persson, Jonatan Vincent, Bertil Lindenfalk, Brant J. Oliver, Andrew D. Smith, Boel Andersson Gäre

**Affiliations:** ^1^ Section of Neurology Department of Internal Medicine County Hospital Ryhov Jönköping Sweden; ^2^ Division of Neurobiology Department of Biomedical and Clinical Sciences Linköping University Linköping Sweden; ^3^ Department of Public Health and Health Care Region Jönköping County Jönköping Sweden; ^4^ Jönköping Academy for Improvement of Health and Welfare School of Health and Welfare Jönköping University Jönköping Sweden; ^5^ Departments of Community and Family Medicine Psychiatry, and the Dartmouth Institute for Health Policy and Clinical Practice at the Geisel School of Medicine at Dartmouth Dartmouth College Hanover New Hampshire USA; ^6^ Multiple Sclerosis Center Dartmouth‐Hitchcock Medical Center and Geisel School of Medicine Dartmouth College Lebanon New Hampshire USA; ^7^ Jönköping Academy for Improvement of Health and Welfare School of Health and Welfare Jönköping University Jönköping and Futurum Region Jönköping County Sweden

**Keywords:** co‐production, cost analysis, healthcare utilization, multiple sclerosis, patient acceptance of health care, primary health care, value architectures

## Abstract

**Background:**

The burden of disease for persons with multiple sclerosis (MS) and society is changing due to new treatments. Knowledge about the total need for care is necessary in relation to changing needs and new service models.

**Objective:**

The aim of this study was to describe the contact patterns for MS patients, calculate costs in health care, and create meaningful subgroups to analyze contact patterns.

**Methods:**

All patients diagnosed with MS at Ryhov Hospital were included. All contacts in the region from January 1, 2018, until September 30, 2019, were retrieved from the hospital administrative system. Data about age, sex, contacts, and diagnosis were registered. The cost was calculated using case costing, and costs for prescriptions were calculated from medical files.

**Results:**

During the 21‐month period, patients (*n* = 305) had 9628 contacts and 7471 physical visits, with a total cost of $7,766,109. Seventeen percent of the patients accounted for 48% of the visits. The median annual cost was $7386 in the group with 10 or fewer visits, compared to $22,491 in patients with more than 50 visits.

**Conclusion:**

There are considerable differences in the utilization of care and cost between patients with MS in an unselected population, meaning that the care needs to be better customized to each patient's demands.

## Graphical abstract

There are considerable differences in care utilization and cost between patients with multiple sclerosis in an unselected population. Less than one‐fifth of the patients had half of the total number of visits, whereas one‐third accounted for 48% of the costs. The median cost was three times higher in the group with more than 50 visits than in the group with less than 11 visits. Our data suggest that the care needs to be better customized to each patient's demands.

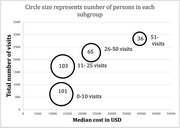

## 1 INTRODUCTION

Multiple sclerosis (MS) can significantly diminish income‐earning ability, impose an extreme financial burden on patients and their families (Owens, 2016), and substantially impact the quality of life and functional capability (Chruzander et al., 2014; Isaksson et al., 2005). Active management, focusing on the person with MS, can minimize disease impact, maximize the quality of life, and espouse a wellness philosophy (Thompson et al., 2018). To provide the best possible care with limited resources, it is crucial to prioritize the MS care that is of the highest value in economic terms and patients’ perceived quality of life, ability to earn income, societal participation, and to decrease the burden on the individual and the social welfare system (Boehmer et al., 2016; Eton et al., 2013).

MS is a costly and complex chronic disease, ranking second behind congestive heart failure in the United States (Adelman et al., 2013; Chen et al., 2017). The annual costs stemming from MS in the healthcare system and society have been estimated to be 27,000 to 68,000$ per patient in the European Union (Kobelt and Pugliatti, 2005; Kobelt et al., 2017). Cost estimates that are currently available have often been based on processes during a limited period of time and are often focused on pharmacological therapy (Melendez‐Torres et al., 2017).

MS is a chronic disease affecting the central nervous system and is treated with drugs targeting the immune system. Cost studies cannot be limited to MS specialty care alone but rather should include services in the entire healthcare system. Patients with MS have been reported to have a high use of care in primary care, neurology, and rehabilitation departments in open care and as inpatients (Chruzander et al., 2015). The reason for the contacts in care is usually expected to be MS, but because data about comorbidities are limited, it could also be due to a condition other than MS (Marrie et al., 2015).

MS therapeutic development has occurred rapidly, thereby creating a greater opportunity for individualized treatment and better outcomes (Piehl, 2014). Therefore, persons with MS may have different needs now than in the era before modern therapy. We aim to identify areas of need in persons living with MS and measure costs in the Swedish healthcare system, including other conditions than MS. A method commonly used for distributing hospital costs is the Swedish version of case costing (CC). The method applies national standards for coding, calculating, and distributing costs to single contacts and/or patients. This is incorporated into a system that calculates costs over time and distributes costs according to national principles (Fitger, 2015).

The aim of this study was to describe the contact patterns for the MS population at a regional hospital in Sweden, serving a defined population in a geographic area, calculate the costs for these persons in the healthcare system, and create meaningful subgroups of contact patterns. This could establish a basis for future studies aiming to improve MS care by addressing care needs specific to each subgroup.

## 2 MATERIALS AND METHODS

Utilization of health care and costs in the healthcare system in Region Jönköping's County (RJC) was analyzed in a retrospective descriptive cross‐sectional study. All persons with a diagnosis of MS (International Classification of Diseases 10 code G35) at the Section of Neurology at the Department of Internal Medicine at Ryhov from January 1, 2018, until September 30, 2019, were included (n = 305). The region is responsible for primary and specialized care but not for nursing homes or care given in patients' homes. Ryhov Hospital is accountable for all persons with MS in three municipalities with a population of 160,800 in the southeastern part of Sweden ((SCB) SS, n.d.). Anonymized data were retrieved from Diver (DivePort version 7.0, Dimensional Insight, Inc.), the information system for analysis and reporting of care given. Data about age, sex, contact types, place of contact, the profession of caregiver, and diagnosis were collected. The number of visits was defined as physical visits, and contacts were defined as physical visits, telemedicine, phone calls, and administrative contacts for prescriptions or certificates.

CC was calculated as specified by the Swedish Association of Local Authorities and Regions (Fitger, 2015). CC is only used for the care given at hospitals. Primary care costs were calculated based on the primary care contact type (visit at the clinic, a home visit, telemedicine, a letter/telephone call) and performer, using average costs for the corresponding combinations in the regions’ primary care.

Costs for prescribed pharmaceuticals in outpatients were calculated from all prescriptions within RJC's medical files during the study period. All costs are presented as annual costs in USD, based on an exchange rate of 1 USD equal to 9.30 SEK, the exchange rate during the study period.

### 2.1 Statistical analysis

Data were initially analyzed using descriptive statistics. Subgroups were analyzed with the aid of graphical analysis and conditional formatting. The patients were first divided into three equally large main groups based on the number of visits. The group with 26 or more visits had a wide range of visits and was therefore divided into two groups, one consisting of two‐thirds of the remaining patients and the last with the one‐ninth of all patients with the most visits. Thereby the groups were corresponding to 3/9, 3/9, 2/9, and 1/9 of all patients.

Data were analyzed using Statistica Version 13.1, Dell Inc. For comparison between the two groups, a *t*‐test was used, and data were presented as the mean and standard deviation (SD). For ordinal data and data not normally distributed, the median and interquartile range (IQR) were given, and the Mann–Whitney *U*‐test was used to compare groups. For the nominal data, the chi2 test was performed. For groups with fewer than five respondents, the analysis was completed with Fisher's exact test. Kruskal–Wallis's analysis of variance (ANOVA) followed by the median test was used for comparisons between more than two groups. Correlations were tested using Spearman rank‐order correlations. Analyses were completed with multiple linear regression. Differences were considered significant at *p* < .05.

### 2.2 Ethics

The study was approved by the Swedish Ethical Review Authority (Dnr 2020–03745).

## 3 RESULTS

### 3.1 Demographics/sampling

Three hundred five patients were included, 199 women with a mean age of 46.8 (SD 14.5) and 106 men with a mean age of 49.1 (SD 14.3; not significant [n.s.] at *p* < .05 level). Patients divided into four groups according to age are presented in Table 1.

**TABLE 1:** Demographics. The number of persons according to age and sex. Number of contacts and physical visits in each age group. Distribution of patients according to subgroups based on age and number of visits during the study period

	Women		Men		Persons	Total share	Contacts	Physical visits	Number of visits in subgroups				**Share of visits in subgroups**			
Age group	*n*	(%)	*n*	(%)	*n*	(%)	*n*	*n*	0–10	11–25	26–50	51+	0–10	11–25	26–50	51+
0–24	15	5%	4	1%	19	6%	506	409	2	12	5	0	11%	63%	26%	0%
25–44	73	24%	34	11%	107	35%	2926	2266	39	32	29	7	36%	30%	27%	7%
45–64	84	28%	55	18%	139	46%	4956	3908	46	44	24	25	33%	32%	27%	7%
65–99	27	9%	13	4%	40	13%	1308	888	14	15	7	4	35%	37%	18%	10%
Total	199	65%	106	35%	305	100%	9696	7471	101	103	65	36				

### 3.2 Contact patterns

#### 3.2.1 All contacts

There were 9628 contacts and 7471 physical visits. The number of contacts and visits at the different clinics is presented in Table 2. Physician's letters or phone contacts were 1178, and 893 were from other staff; 667 of these were from primary care.

**TABLE 2:** Annual cost and cost of prescriptions in different age groups

	Persons	**Total cost**		**Prescriptions**	
Age‐group	*n*	Median	Interquartile range (IQR)	Median	IQR
0–24	19	8998	5984–15,307	140	52–201
25–44	107	10,279	5887–22,957	601	84–9502
45–64	139	12,839	6374–21,125	2064	466–9970
65–99	40	9291	4152–10,847	983	331–2358

*Note*: Costs are presented in USD.

Four percent of the patients accounted for 15% of the contacts, and 19% accounted for 49% of healthcare contacts. Forty‐six percent of the patients had only 16% of the contacts. There was no significant trend that the number of visits increased as patients got older (Spearman *ρ* 0.037, n.s. at *p* < .05 level).

#### 3.2.2 Physical visits

The most common main diagnosis for outpatients was MS in 2622 visits. The number of visits at the different clinics ranged from 16 to 2562, with most in primary care; see Table 2. Seventeen percent of the persons accounted for 48% of the visits, and 49% of the patients with the least number of visits accounted for 16%. Ninety percent of the patients had visited at least three clinics (primary care considered one clinic), and 27% had visited seven or more clinics. The mean number of clinics that patients had visited was 4.4 (SD 2.0), of which 39% were to a physician. The number of visits to different professionals is presented in Table 3.

**TABLE 3:** Contact, visits, and costs for MS patients in different specialties

					**Mean number of visits in subgroups based on the total number of visits**				**Annual costs**		
Clinic	Persons with visits	Percent of persons	Number of contacts	Number of physical visits	0–10	11–25	26–50	51+	Prescriptions*	CPP	Total cost
Neurology	305	100%	2103	1441	2.6	4.7	6.1	8.3	$163,069	$1,266,883	$2,903,953
Primary care	261	86%	3664	2599	2.0	5.7	11.5	29.3	$87,209	$242,609	$329,817
Rehab center	145	48%	1230	1150	0.6	1.7	5.8	15.1	$ –	$103,208	$103,208
Rehabilitation medicine	54	18%	906	875	0.0	0.9	4.3	13.9	$4198	$394,690	$398,888
Ophthalmology	82	27%	259	237	0.9	0.9	1.0	1.6	$1867	$29,711	$31,578
Surgery	68	22%	215	169	0.2	0.3	0.9	1.6	$3507	$165,699	$169,207
Gynecology	99	32%	297	256	0.4	0.7	1.7	1.0	$4617	$41,348	$45,965
Psychiatry	14	5%	144	112	0.0	0.2	0.3	1.9	$2989	$56,604	$59,593
Orthopedics	42	14%	129	109	0.0	0.4	0.5	0.8	$828	$59,781	$60,609
Oncology	9	3%	136	101	0.0	0.1	0.5	1.4	$2850	$64,190	$107,040
Urology	37	12%	145	90	0.1	0.3	0.5	0.8	$1862	$47,635	$49,497
Dermatology	33	11%	94	85	0.1	0.3	0.6	0.3	$1959	$10,459	$12,418
Ear, nose, throat	36	12%	79	75	0.1	0.3	0.2	0.6	$149	$14,126	$14,275
Internal medicine/geriatrics	19	6%	174	121	0.0	0.0	0.7	2.0	$5415	$106,412	$111,827
Other	18	6%	53	51	0.0	0.2	0.3	0.2	$4234	$35,659	$39,893
**Total**	**305**		**7525**	**7471**	**6.3**	**17.0**	**35.1**	**72.7**	$1,798,753	$2,639,015	$4,437,768

* Costs for drugs given at the day‐care unit are included in the CPP.

#### 3.2.3 Hospitalizations

Seventy‐eight persons had 156 hospitalizations. The most common department was neurology, 65; followed by surgery, 25; urology, 14; internal medicine and geriatrics, 14; rehabilitation medicine, nine; and psychiatry, nine. MS or demyelinating disease of the central nervous system was the primary diagnosis in 37 of the hospitalizations; nine were due to urinary tract infection, eight were due to nephritis, four were alcohol‐related disorders, and four were erysipelas. The patient with the most hospitalizations had nine episodes, mainly due to MS and epileptic seizures.

### 3.3 Costs

The total cost of the MS patient group during the studied interval was $7,766,109, corresponding to an annual cost of $4,437,777 with a median cost of $9937 (range $222–109,723, IQR $5923–20,400) per patient. There was no difference in cost between women and men (median $9937 for women, and $12,760 for men, n.s. at *p* < .05 level). Costs in the age groups are presented in Table 2. There was no linear correlation between patients' age and cost (*ρ* = −0.06, n.s. at *p* < .05 level). In a Kruskal–Wallis ANOVA between the age groups, the difference was significant (*p* < .01), but the only significant differences were between the 65 or older group and the 25–44 and 45–64 groups. As expected, the cost increased with a higher number of visits (*ρ* = 0.36, *p* < .05). The total annual CC without cost for prescriptions was $2,639,015. The costs at different clinics are presented in Table 3.

### 3.4 Prescriptions

There were 4079 prescriptions at a total annual cost of $1,798,753. The median cost was $1098 (range $0–53,412, IQR 199–8444. The highest number of prescriptions per year were paracetamol *n* = 121, zopiclone *n* = 85, dimethyl fumarate 55, oxycodone 53, and gabapentin *n* = 51. The median cost of prescriptions was $840 (range: 0 −35,584) for men and $1354 (range 0–53,412) for women (n.s. at *p* < .05 level). The median cost in the different age groups is presented in Table 2. Kruskal–Wallis ANOVAs showed that the differences between groups were significant (*p* < .001).

Six drugs had yearly costs exceeding $60,000 dimethyl fumarate $586,036, fingolimod $480,732, glatiramer acetate $244,109, interferon beta‐1a $ 183,484, interferon beta‐1 b $91,352, and teriflunomide $62,171. Pegylated interferon beta‐1a, encorafenib, binimetinib, gabapentin, and sodiumoxybate all had costs exceeding $10,000. Encorafenib and binimetinib are used as treatments for malignant melanoma.

### 3.5 Subgroups based on number of visits

Subgroups based on the number of visits were created. The results are presented in Tables 2, 3, 4. The group with more than 50 visits was significantly older than patients with 25 to 50 visits (*p* = .019). There were no other differences in age between groups.

**TABLE 4:** Visits at different professions and annual costs in subgroups based on the total number of visits

		**Mean number of visits per profession (SD)**										**Median cost in USD (IQR)**		
**Subgroups based on the number of visits**	**Total number of visits**	**Physician**	**Nurse**	**Physiotherapist**	**Occupational therapist**	**Psychologist**	**Counselor**	**Midwife**	**Optician/orthoptician**	**Speech therapist**	**Other**	**Prescriptions**	**Case costing**	**Total cost**
0–10 (*n* = 101)	622	2.9	2.1	0.4	0.3	0.0	0.1	0.3	0.0	0.0	0.1	$4948	$1604	$7386
		(1.8)	(2.0)	(0.8)	(0.6)	0	(0.6)	(0.6)	(0.1)	(0.2)	(0.6)	(104–13,110)	(965–4774)	(4660–15,624)
11–25 (*n* = 103)	1730	7.8	5.2	1.6	0.7	0.3	0.4	0.5	0.2	0.0	0.2	$721	$5677	$7648
		(4.3)	(4.3)	(2.2)	(1.3)	(1.3)	(1.7)	(1.3)	(0.5)	(0.4)	(0.5)	(145–6811)	(3725–7253)	(5515–16,095)
26–50 (*n* = 65)	2282	14.4	8.8	5.6	1.9	1.7	1.0	1.2	0.1	0.2	0.1	$1338	$8466	$13,157
		(8.2)	(8.7)	(7.5)	(2.7)	(4.1)	(3.1)	(3.2)	(0.6)	(1.2)	(0.7)	(239–6997)	(6005–17,185)	(9418–21,463)
51+ (*n* = 36)	2837	24.6	29.3	17.1	1.0	2.1	1.8	0.2	0.3	0.1	1.9	$1151	$18,539	$22,491
		(14.7)	(22.8)	(21.0)	(1.8)	(6.5)	(4.2)	(0.4)	(0.5)	(0.5)	(4.6)	(571–3844)	(13,099–28,696)	(15,161–33,862)
Total number of visits	7471	2901	2370	1192	257	211	187	171	40	25	117			

**FIGURE 1:**
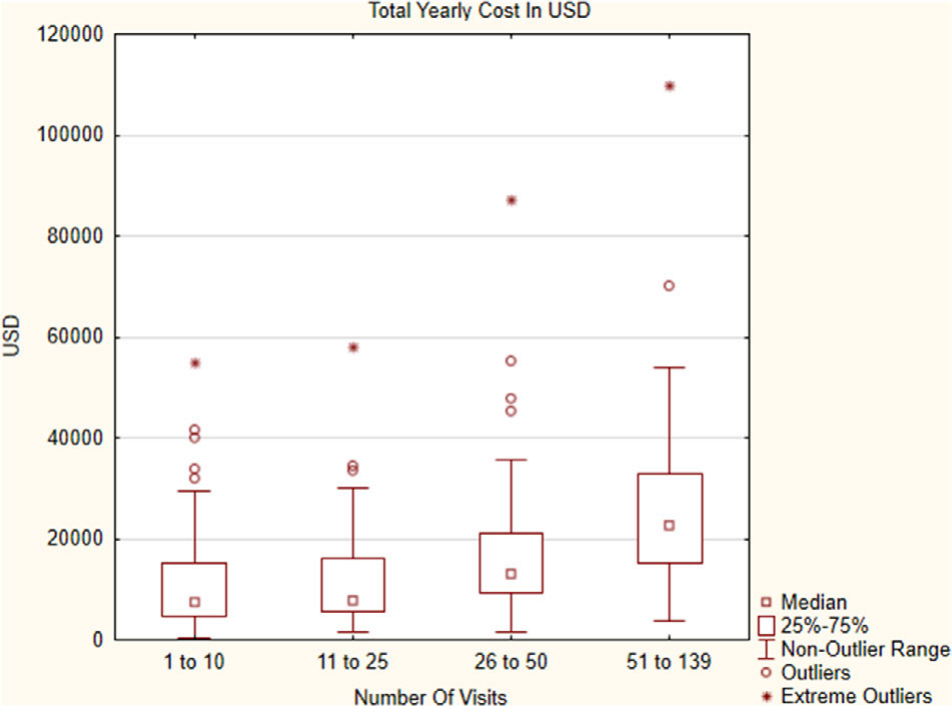
Median yearly cost of health care in USD in subgroups based on the number of visits

**FIGURE 2:**
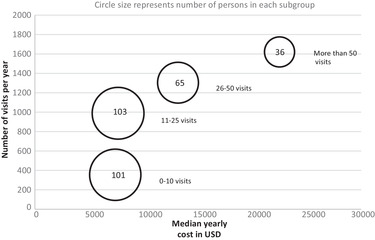
Median annual cost in USD, number of visits per year, and the number of persons in subgroups based on the total number of visits are presented

The cost for prescriptions was not statistically significant between the subgroups; however, CC increased with an increasing number of visits (*p* < .001 for all comparisons except 26 to 50 visits vs. the 51+ group; see Table 3). The total annual cost in the respective subgroups was $1,129,994, $1,186,286, $1,135,945, and $985,553. Figure 1 presents the subgroups' median values and IQR (*p* < .001 for all comparisons between separate groups, except 1–10 vs. 11–25 and 26–50 vs. 51–131 visits). The median annual cost in USD, the number of visits per year, and the number of persons in subgroups based on the total number of visits are presented graphically in Figure 2.

## 4 DISCUSSION

We found considerable differences in care utilization and costs between persons in an unselected MS population, summarized in Figure 2. Less than one‐fifth of the persons had half of the total number of visits. Our data do not provide an easy explanation, but overutilization of care has been found in other groups of patients (Knapp et al., 2021). There was no correlation between age and number of visits when tested with linear regression. The cost was lowest in patients older than 65 years, probably due to fewer persons on expensive disease‐modifying treatments, which can be confirmed with data from the MS registry, where only 15 of the patients 65 years or older were on treatment and then mostly on interferons (Swedish Neuro Registries, n.d.).

CC can help answer questions such as which group of patients costs the most at a clinic or the cost of a particular service for a specific group of patients (Tan et al., 2014). It makes it possible to look at individual patients to follow the cost they create. Since the calculations are based on each event, it is possible to measure change over time, both costs for specific patients or processes, and to better evaluate interventions in economic terms. CC is also an excellent tool for making simulations.

We found that the variation in the number of visits is driven mainly by rehabilitation, with an increasing number of visits to the physiotherapist in each subgroup based on the number of visits. In the group with up to 10 visits, only 8% of the visits were to a physiotherapist, while for the group with more than 50 visits, the portion increased to 27%. However, there was no increase in the number of visits to an occupational therapist. Compared to previous reports, one crucial difference in our material is that hospitalizations, especially in rehabilitation medicine, are far less common (Chruzander et al., 2015). This is most likely a combined result of improvements in treatment and a general change in rehabilitation medicine from hospitalizations to different forms of outpatient rehabilitation.

To get a better understanding, patients were divided into four subgroups based on the number of visits (see Figure 2). Patients in the groups with a higher number of visits had higher total costs, but the cost for prescriptions was not affected by the subgroup. The reasons for a patient to have many visits and high costs have to be further explored. One explanation could be that there is a limit to how much a patient needs to pay per year in the Swedish system, that is, after you reach that limit, care for the rest of the year is free for the individual, which might affect both patients and caregivers in decisions about visits. The ability to cope with a chronic disease and comorbidities might be an essential factor (Strober, 2017). The main number of visits for patients with many visits was related to MS, either at neurology, rehabilitation, or primary care clinics, and might be attributed to disease activity or disabilities that have not required new expensive medications.

The most significant cost component for individuals with MS is disease‐modifying therapies, representing, for example, 53% of the MS‐associated cost in Germany (Müller et al. 2020). The rising cost of MS treatment is a well‐known problem, with the cost of most disease‐modifying drugs in the United States exceeding $70,000 a year (Hartung, 2017). Sweden has a high use of off‐label rituximab (Salzer et al., 2016), with a lower annual cost of approximately $1300. According to the Swedish National Quality Registry for MS, 98 of the 305 patients in this study received treatment with rituximab in March 2019 (Swedish Neuro Registries, n.d.). The rituximab cost is included in CC in our study, as the treatment was given at the hospital.

This study differs from previously published studies about costs in MS, as all healthcare costs were retrieved from the region responsible for all care in the area. A frequent problem in previous studies is which costs should be attributed to MS. One important finding is that oncological therapies were among the most expensive drugs, even in this relatively young patient cohort. A recent community‐based study from Spain found an increased risk for stroke, epilepsy, bipolar disorder, and depression among MS patients (Cardenas‐Robledo et al., 2021), underlining the need to consider care and costs other than those directly attributed to MS.

Lower coping capacity, impaired manual dexterity, and activity of daily living dependency at baseline, together with progress in MS disability, predicted a higher use of care in a Swedish 10‐year population‐based study (Chruzander et al., 2015). To provide MS care that creates maximal value for the patient individually and for the group, initiatives and research programs should improve their ability to assess and report meaningful patient outcomes in many dimensions, including costs. One model for such balanced measures is the “Value Compass.” We are part of the COproduction VALUE creation in healthcare service (CO‐VALUE) study, which aims to find novel ways to use resources in the best way and co‐produce and co‐design care with patients and the network around the patients (Oliver et al., 2020).

### 4.1 Limitations

A significant limitation is that the data were retrieved from the hospital's reporting system without MS‐specific data, and we did not have ethical approval to use personal identification numbers or other databases. This means that we cannot relate our findings to the disease duration, clinical course, patient symptoms, or the Expanded Disability Status Scale, which in other studies have been found to affect costs (Kobelt et al., 2017; Müller et al., 2020). This will be studied as our next step.

The data were retrieved from a single hospital, and the number of patients was limited; therefore, one should be careful to generalize the results. However, the study is population‐based, including all patients with MS in the geographic area, since RJL is responsible for all health care of persons living in the region.

The results for significance testing are presented without correction for multiplicity testing; hence, *p*‐values greater than .01 should be interpreted with caution.

The contact data reported here represent the most conservative estimates, as all contacts were not registered, but using CC does not affect the total cost, as these costs are included in the overhead cost, and the cost is allocated to the next visit. Phone calls may represent a surrogate marker of unmet needs, and the more calls that come in, the more nursing utilization there is, which then increases related costs allocated to subsequent care visits.

### 4.2 Future directions

Our study raises many questions about population‐level variation in care utilization in Sweden. Healthcare systems in Sweden and elsewhere often aim to move a person with a chronic disease from the group needing specialist care to primary care and to involve peer support from networks and, when, if possible, to self‐care (Suutari et al., 2019).

In a diverse population of patients with MS, it might be more cost‐efficient to design care for the individual person by adapting the service configuration and use of telemedicine when appropriate. We are part of an international collaboration exploring co‐production (Oliver et al., 2020), and based on that, value configurations and service offerings will be further evaluated through qualitative inquiry with patients from the four subgroups. The results will be evaluated using Fjeldstad's theories about the value configurations of the value chain, value shop, and value network health in care (Fjeldstad et al., 2020).

According to the Batalden co‐production of health, “the interdependent work of users and professionals who are creating, designing, producing, delivering, assessing, and evaluating the relationships and actions that contribute to the health of individuals and populations. At its core are the interactions of patients and professionals in different roles and degrees of shared work” (Batalden, 2018). Evaluation and outcomes related to co‐production can be challenging (Voorberg et al., 2014). It has been argued that outcome measures such as patient satisfaction may overlook the real value created for patients and staff and are too narrow in their construct. Future studies of coevaluation in health care should include clinical outcomes, patient‐reported outcomes from multiple service processes and outcomes, and cost‐effectiveness (Clarke et al., 2017). We would add the lived experiences of persons living with MS in a deeper sense than just traditional questioning. A model for co‐production value in MS is being developed to improve care (Smith et al., 2020). In addition, we intend to study the effect of using a national initiative on making co‐designed care contracts with patients.

## 5 CONCLUSION

Persons with MS are very diverse and have very different needs in relation to the healthcare system. Understanding care needs and utilization patterns can inform targeted co‐production and co‐design approaches to meet the specific care needs of identified individuals and subgroups and improve outcomes in a broad sense.

## CONFLICT OF INTEREST

The authors declare that there is no conflict of interest.

### PEER REVIEW

The peer review history for this article is available at https://publons.com/publon/10.1002/brb3.2582

## Data Availability

The data that support the findings of this study are available from the corresponding author upon reasonable request.
